# Organic Synthesis of New Secosteroids from Fucosterol, Its Intestinal Absorption by Caco-2 Cells, and Simulation of the Biological Activities of Vitamin D

**DOI:** 10.3390/md21100540

**Published:** 2023-10-17

**Authors:** Shiro Komba, Megumi Hase, Eiichi Kotake-Nara

**Affiliations:** Institute of Food Research, National Agriculture and Food Research Organization, 2-1-12 Kannondai, Tsukuba 305-8642, Ibaraki, Japan; skomba@affrc.go.jp (S.K.); hasem120@affrc.go.jp (M.H.)

**Keywords:** Caco-2 cells, fucosterol, intestinal absorption, mixed micelles, PASS, secosteroid, vitamin D

## Abstract

We previously examined the cellular uptake of six types of vitamin D in human intestinal Caco-2 cells. Since vitamins D_5_–D_7_ were commercially unavailable, we synthesized these compounds organically before studying them. This process led us to understand that new secosteroids could be generated as vitamin D candidates, depending on the sterol used as the starting material. We obtained two new secosteroids—compounds **3** and **4**—from fucosterol in the current study. We investigated the intestinal absorption of these compounds using Caco-2 cells cultured in Transwells and compared the results with vitamin D_3_, a representative secosteroid. The intestinal absorption of compound **4** was comparable to that of vitamin D_3_. Compound **3** showed similar uptake levels but transported about half as much as vitamin D_3_. These compounds demonstrated intestinal absorption at the cellular level. Vitamin D is known for its diverse biological activities manifest after intestinal absorption. Using PASS online simulation, we estimated the biological activity of compound **3**’s activated form. In several items indicated by PASS, compound **3** exhibited stronger biological activity than vitamins D_2_–D_7_ and was also predicted to have unique biological activities.

## 1. Introduction

Vitamin D is an essential nutrient crucial for the intestinal absorption of bone-building minerals like calcium and phosphorus. First isolated as an anti-rickets factor in the early 1930s from UV-irradiated ergosterol products, vitamin D_2_ has a characteristic secosteroid structure and is classified as a steroid hormone [1,2]. Vitamin D plays a multi-faceted role in human health. Specifically, its blood levels are associated with a range of health conditions and risks, including maternal and child health [3,4], reproductive functions [5,6,7], physical performance [8], renal function [9], and sleep quality [10], as well as diseases like cancer [11,12,13,14], lung disease [15,16,17,18,19], vascular disease [20,21,22], metabolic syndrome [23,24], dementia [25,26], psychiatric disorders [27,28,29,30], viral diseases [31,32,33,34,35] including COVID-19, and intractable diseases like myofibromyalgia [36]. Furthermore, adequate vitamin D levels can reduce chronic inflammation, a known trigger for various serious diseases [37,38]. Maintaining high levels of vitamin D may help prevent some diseases.

Unfortunately, vitamin D deficiency is common [39,40]. For example, 98% of 5518 Japanese individuals tested were found to be vitamin D deficient [41]. UVB irradiation enables the biosynthesis of vitamin D_3_ from 7-dehydrocholesterol in sebum. This biosynthesis is influenced by season, latitude, and age. In certain geographic locations like Sapporo (Japan), sun-induced vitamin D_3_ biosynthesis is nearly impossible during winter [42]. The skin’s ability to produce 7-dehydrocholesterol also diminishes with age [43]. Excessive UV exposure has adverse health effects like intraocular melanoma and cataracts [44]. That is why it is probably best to obtain vitamin D from food as much as possible. However, dietary sources of vitamin D are limited—fish and shellfish for vitamin D_3_, and mushrooms for vitamins D_2_ and D_4_ [45]—as it is rarely found in the edible parts of fruits and vegetables (in contrast to inedible parts like leaves of tomatoes, which are typically rich in vitamin D_3_) [46,47]. This makes it challenging to maintain adequate levels, especially for vegetarians and vegans. We found that we were able to create new vitamin D compounds via the 7-dehydro form, depending on the type of sterol used. Because we had no commercial source of vitamins D_5_–D_7_, we organically synthesized vitamins D_5_–D_7_ for this study using commercially available plant sterols—specifically, β-sitosterol, campesterol, and stigmasterol—as starting materials [48].

A prior study sought to determine if it was possible to prevent a vitamin D deficiency by increasing the intake of vitamins D_2_–D_7_ [49]. For vitamin D to work in the body as a nutrient or functional ingredient, it must first be absorbed through the intestinal tract. Comparison of vitamin D absorption in the intestinal model cell Caco-2 showed no significant differences among vitamins D_2_–D_7_. This suggests that not only vitamins D_2_ and D_3_ but also vitamins D_4_–D_7_ can be absorbed in the intestinal tract, at least at the in vitro cellular level.

**Figure 1:**
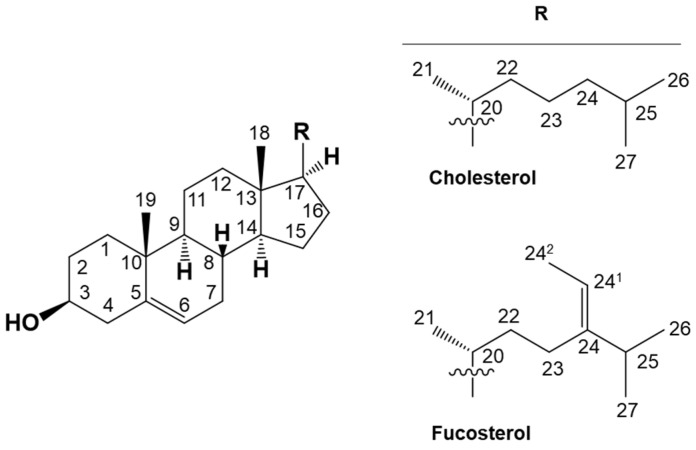
Chemical structural formulas depicting cholesterol, a representative sterol, and fucosterol, utilized as the initial material for the organic synthesis of novel secosteroids. Numbers indicate carbon positions. Cholesterol and fucosterol have a common cholestane skeleton. “R” indicating a different side chain binds to this skeleton.

We synthesized a new secosteroid using fucosterol as the starting material in the present study (Figure 1). We evaluated its intestinal absorption in Caco-2 cells, comparing it with that of vitamin D_3_. Vitamin D absorbed in the intestinal tract does not exhibit any function by itself. While vitamin D requires metabolic activation in the liver and kidney to function, the new secosteroid may follow the same activation pathway. This activated form’s nutritional and functional properties were estimated using a Prediction of Activity Spectra for Substances (PASS) online simulation, like in our previous study [49]. The possible natural occurrence of this new secosteroid is also discussed.

## 2. Results

### 2.1. Organic Synthesis

**Figure 2:**
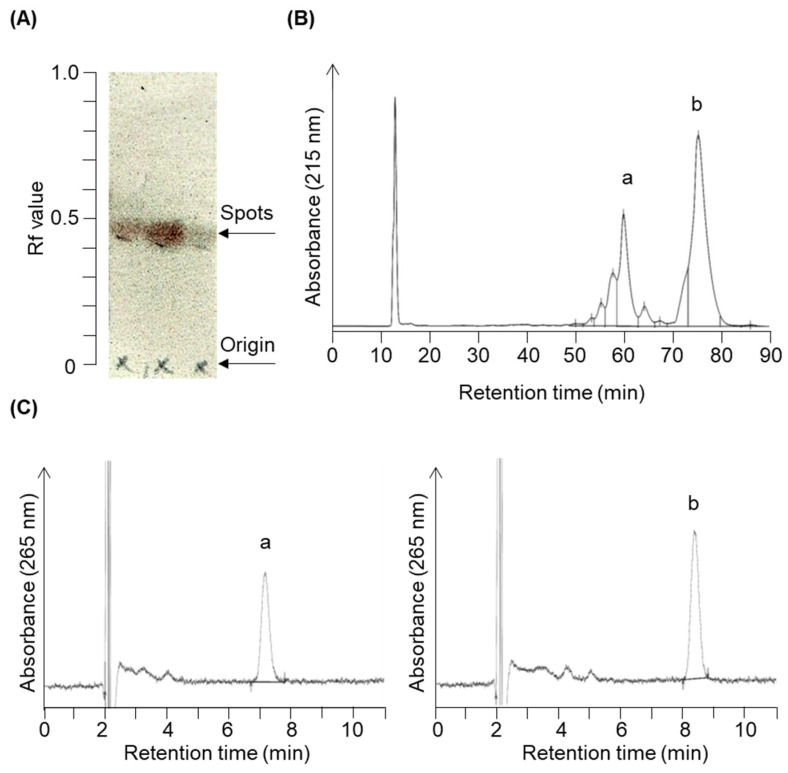
Chromatogram profiles illustrating the chromatographic separation of target compounds **3** and **4**. The reaction solution was concentrated following the reaction shown in Figure 7e. (**A**) The concentrated product contained a constituent with an Rf value of 0.43 upon expansion in acetic ether/normal hexane (1:4, *v*/*v*) during silica gel thin-layer chromatography. Column chromatography eluted and recovered this fraction in acetic ether/normal hexane (3:7, *v*/*v*). (**B**) Further purification using reverse-phase HPLC (condition 1) yielded two prominent peaks around 60.1 min (peak a) and 75.4 min (peak b). (**C**) HPLC chromatograms (condition 2) of purified peak a (**left panel**, compound **4**) and peak b (**right panel**, compound **3**).

**Figure 3:**
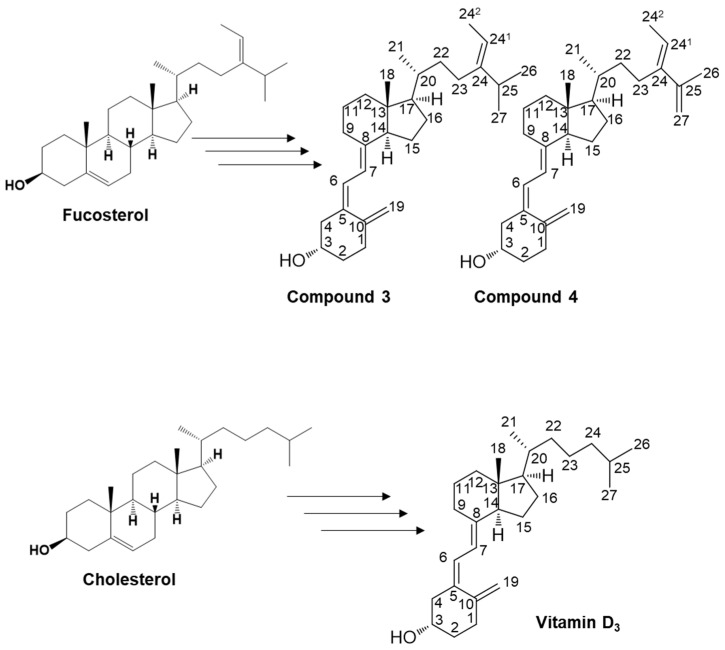
Illustration of the potential pathways for the organic synthesis of secosteroids for subsequent NMR analysis. The carbon position numbering corresponds to the structural configuration of compounds **3** and **4**, and vitamin D_3_. This pathway represents a plausible route for compounds featuring a backbone similar to cholesterol in organic synthesis. Such an in vivo pathway has yet to be identified in humans.

We have organically synthesized novel secosteroids, using fucosterol as the initial compound. As depicted in Figure 2, the final high-performance liquid chromatography (HPLC) analysis isolated two pure compounds—compounds **3** and **4**. We used various nuclear magnetic resonance (NMR) techniques to examine their structures, including ^1^H-NMR, ^13^C-NMR, COSY-NMR, HSQC-NMR, HMBC-NMR, and NOESY-NMR. The peak (b) at 75.4 min (Figure 2B) was identified as the desired compound **3** (Figure 3). The NOESY-NMR spectrum exhibited cross-peaks from H-24^1^ (5.19 ppm) to Me-26 or 27 (0.979 or 0.983 ppm), another cross-peak to Me-24^2^ (1.57 ppm), and additional ones to H-25 (2.23 ppm). These observations establish the geometry of the C-24-C-24^1^ double bond as an E configuration, confirming its identity as target compound **3**. Conversely, the peak (a) at 60.1 min, expected to possess an extra double bond due to a two-hydrogen decrease in molecular weight, was confirmed as compound **4** using intricate NMR analyses. In the NOESY-NMR spectrum, cross-peaks emerged from H-24^1^ (5.63 ppm) to Me-24^2^ (1.71 ppm), as well as from Me-26 (1.87 ppm) to H-27a (4.86 ppm), and H-27b (4.96 ppm) to H-23a (2.08–2.21 ppm) and H-23b (2.26–2.43 ppm). These findings establish the E-configuration of the C-24–C-24^1^ double bond and the existence of the double bond between C-25 and C-27 in compound **4**.

### 2.2. Cell Biochemistry

We evaluated the intestinal absorption of compounds **3** and **4**, and then compared both compounds to vitamin D_3_, a representative secosteroid.

#### 2.2.1. Levels of Micellar Secosteroid Remaining

**Figure 4:**
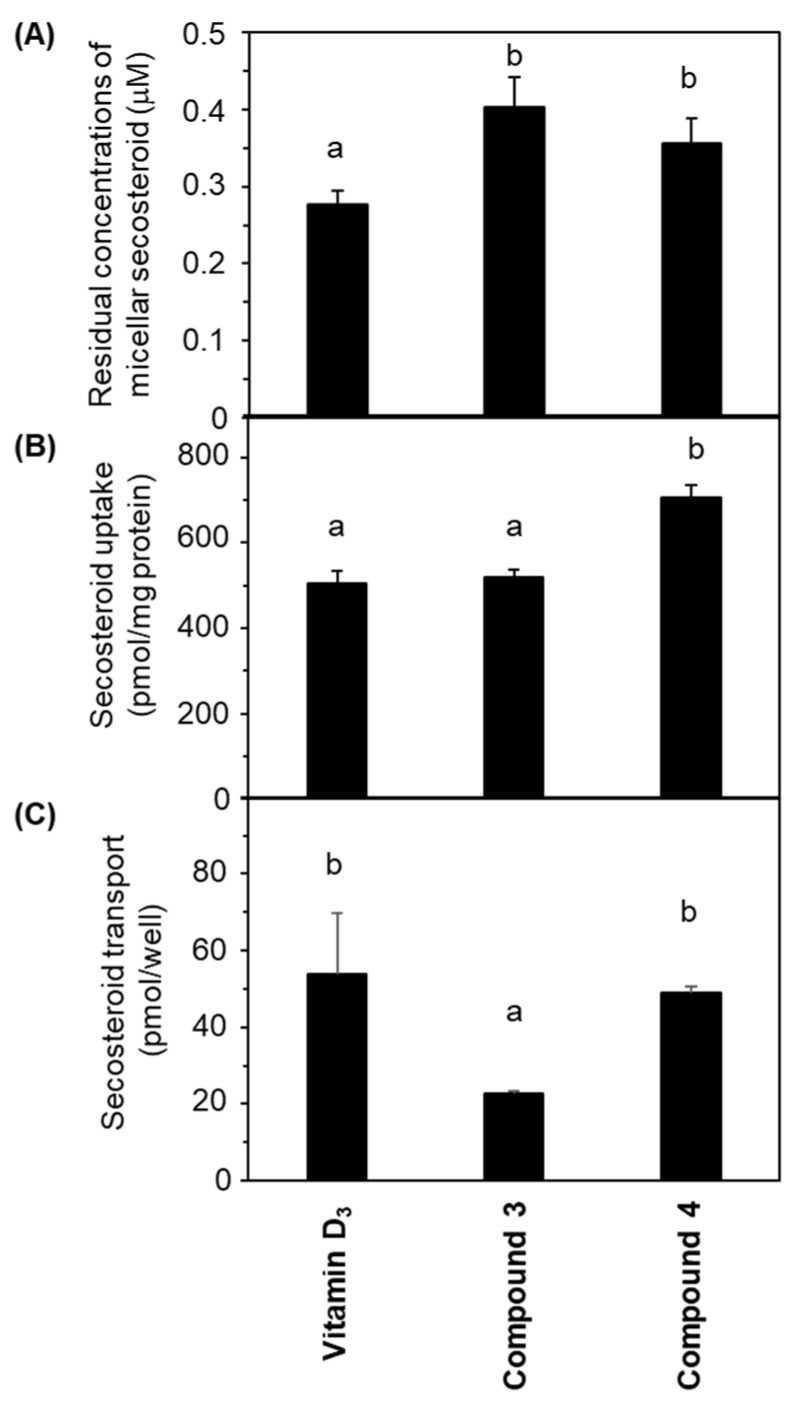
Comparative depiction of secosteroid intestinal absorption using differentiated Caco-2 cell monolayers. Cells were exposed to mixed micellar secosteroid in Transwell plates for 24 h. (**A**) Remaining concentrations of secosteroid in the mixed micelles (apical side). (**B**) Uptake of secosteroid by differentiated Caco-2 cells forming the tight monolayer. (**C**) Quantities of secosteroid transported from the tight monolayers into the basolateral medium. Data represent means ± standard deviations from four wells in a Transwell plate for a single experiment. Replicate experiments exhibited consistent trends. Values sharing a common alphabet were statistically indistinct, as determined by the Mann–Whitney U-test (*p* < 0.05).

Figure 4A illustrates the concentrations of micellar secosteroid remaining on the apical side following a 24 h incubation period. The initial concentration of micellar secosteroids was 1 μM, and more than 30% of them remained. Compounds **3** and **4** exhibited significantly higher concentrations than vitamin D_3_. No discernible difference was observed between compounds **3** and **4**.

#### 2.2.2. Uptake of Secosteroid by Differentiated Caco-2 Cells

Figure 4B presents the amount of secosteroids taken up by the cells from the apical side after 24 h of incubation. Compound **4** had the highest uptake; compound **3** exhibited a similar uptake to vitamin D_3_.

#### 2.2.3. Transport of Secosteroid by Differentiated Caco-2 Cells in Tight Monolayer Configuration

Figure 4C demonstrates the secosteroids secreted from the cells into the basolateral side following a 24 h incubation. No significant deviation was observed between vitamin D_3_ and compound **4**. The transported amount of compound **3** was approximately half of these.

No evidence of leakage due to operational errors in the tight monolayer setup was observed from measuring the concentration of phenol red in the basolateral medium.

### 2.3. Estimating the Biological Activity of Secosteroids

**Figure 5:**
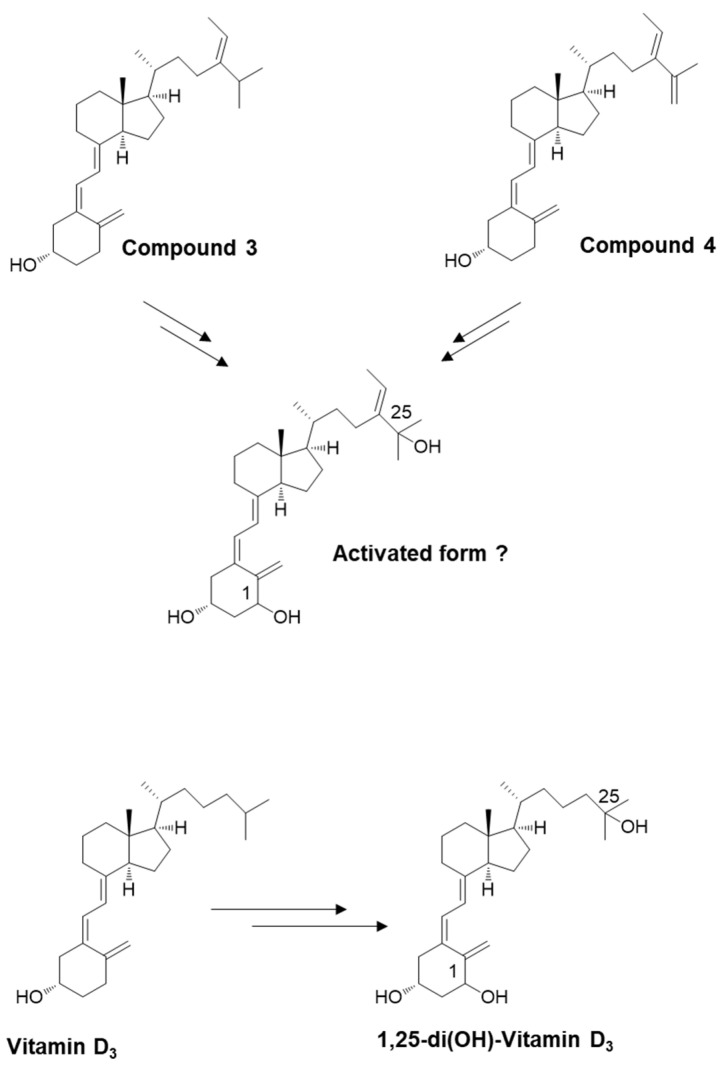
Schematic depicting the potential for converting newly synthesized compounds into their activated forms. The introduction of an OH group at position 25 (in the liver) and position 1 (in the kidney). Similar to vitamin D_3_, compound **3** appears likely to undergo conversion to an activated state.

**Figure 6:**
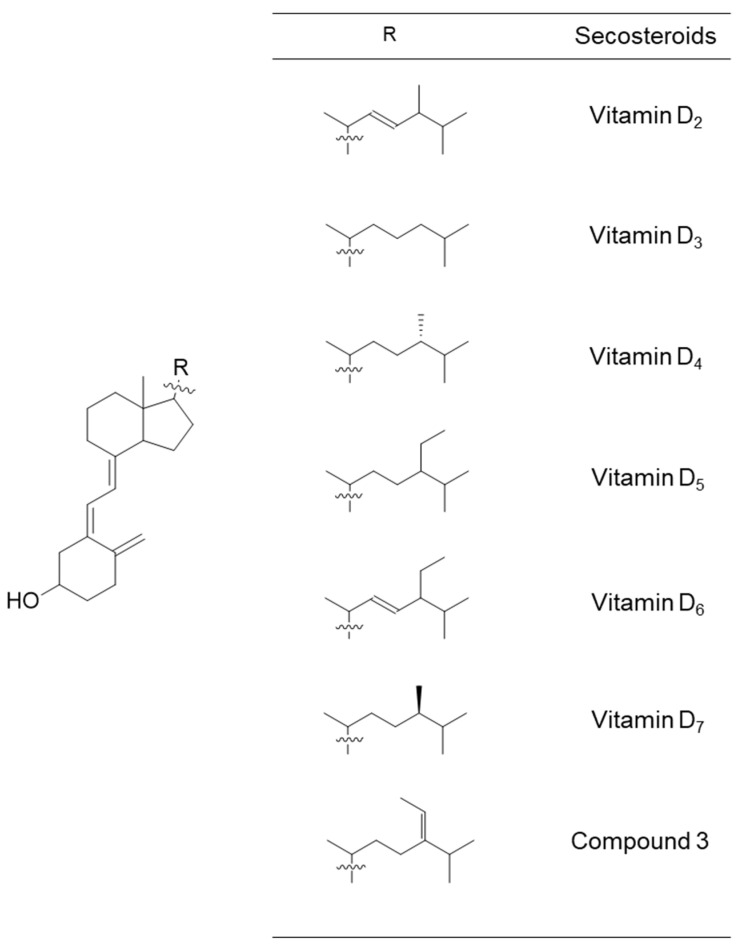
Chemical structural formulas of vitamins D_2_–D_7_ and compound **3**. These secosteroids were transformed into the structural formula of 1,25-di(OH) for input into the PASS online tool to estimate their biological activity.

We used the secosteroid-activated form, featuring hydroxyl groups at positions 1 and 25. Considering the potential commonality of the activated form for compounds **3** and **4** and the uncertainty surrounding the activation of position 25 in compound **4**, we assumed the activated form of compound **3** (Figure 5). The estimated and observed activities of each compound were generated and compared to vitamin D_3_, which is representative, but also vitamins D_2_, D_4_, D_5_, D_6_, and D_7_ (Figure 6).

#### 2.3.1. PASS Online Simulation Part 1

Table 1 delineates relevant items related to vitamin D nutrition, including bone health and calcium regulation. While vitamins D_2_ and D_3_ excelled in several items, vitamins D_5_ and D_6_ also demonstrated high potency, particularly in the activities vitamin, calcium regulator, vitamin D-like, and vitamin D receptor agonist. Vitamin D_4_/D_7_ and compound **3** exhibited lower Pa values than other vitamin D types, yet exceeded 0.9 for anti-osteoporotic, bone disease treatment, and vitamin activities.

#### 2.3.2. PASS Online Simulation Part 2

Table 2 lists nine items related to skin and tumors. Vitamin D_2_ had two items (apoptosis agonist and antileukemic) with the highest Pa value and three items (antipsoriatic, dermatologic, and antineoplastic) with the second-highest Pa value. Vitamin D_3_ had the highest Pa values with two items (anti-eczematic and antipruritic). Vitamin D_5_ had one item (adenomatous polyposis treatment) with the highest Pa value and three items (antipruritic, antileukemic, and chemopreventive) with the second-highest Pa value. Vitamin D_6_ had three items with the highest Pa value (antipsoriatic, dermatologic, and antineoplastic) and one item (apoptosis agonist) with the second-highest Pa value. Vitamin D_4_/D_7_ had fewer items with higher Pa values than the other secosteroids; only one item (adenomatous polyposis treatment) demonstrated the second-highest Pa value. In compound **3**, there was one item (chemopreventive) with the highest Pa value and one item (anti-eczematic) with the second-highest Pa value.

#### 2.3.3. PASS Online Simulation Part 3

Table 3 also lists nine items that were mainly related to inflammation and immunity. Vitamin D_2_ had two items (multiple sclerosis treatment and anti-parkinsonian, rigidity relieving) with the highest Pa value. Vitamin D_3_ had the highest Pa values with four items [respiratory analeptic, analeptic, anti-inflammatory, and antidiabetic (type 1)] and the second-highest Pa values with one item [anti-viral (rhinovirus)]. Vitamin D_5_ had four items [respiratory analeptic, analeptic, antidiabetic (type 1), and anti-fungal] with the second-highest Pa value. Vitamin D_6_ had two items (multiple sclerosis treatment and anti-parkinsonian, rigidity relieving) with the second-highest Pa value.

Compound **3** had three items [polarization stimulant, anti-viral (rhinovirus), and anti-fungal] with the highest Pa value and one item (anti-inflammatory) with the second-highest Pa value. Notably, there was an item (polarization stimulant) that is not found in vitamins D_2_–D_7_ and is the only output, with a Pa value of 0.87.

## 3. Discussion

Common forms of vitamin D include D_2_ and D_3_. Beyond these, limited information exists about vitamins D_4_–D_7_, initially reported in 1942 [50,51,52,53]. Naturally, vitamins and minerals are ineffective unless absorbed by the body. In a previous study, we investigated the intestinal absorption of vitamins D_2_–D_7_ using human intestinal model cells [49]. In that study, vitamins D_5_–D_7_ were organically synthesized [48] because they were commercially unavailable at the time of the study. For vitamins D_2_ and D_3_, commercial products like ergosterol and 7-dehydrocholesterol served as starting materials. However, for vitamins D_5_–D_7_, no commercial 7-dehydrosterols were available. Instead, we used plant sterols such as β-sitosterol, campesterol, and stigmasterol as starting materials. Through that research, we realized the potential to create novel forms of vitamin D from sterols.

In the present study, we performed the organic synthesis of secosteroids, which could be new vitamin D candidates. Compounds **3** and **4** were synthesized starting from fucosterol. Compound **4** contains an extra double bond. The rationale is as follows: The hydroxy group of fucosterol was first acetylated, followed by the Wall–Ziegler reaction [54,55,56,57] to brominate the allyl position. This bromination likely occurred at both the 7 and 25 positions of the fucosterol. Subsequent treatment with tetrabutylammonium fluoride led to synthesizing the desired compound with a ∆^5,7^-diene and another compound containing a ∆^5,7^-diene and an additional double bond between C25 and C27. To the best of our knowledge, both compounds **3** and **4** are novel secosteroids.

The critical step in secosteroid synthesis is the introduction of a ∆^5,7^-diene into the sterol backbone. In insects, cholesterol 7,8-dehydrogenase (DAF-36/neverland) converts cholesterol to 7-dehydrocholesterol [58]. This enzyme is not commercially available, prompting us to opt for organic synthesis. Unfortunately, no equivalent enzyme has been identified in mammalian genomes [58].

For fat-soluble nutrients like vitamin D to function, they must be absorbed in the intestinal tract post-ingestion. Forms of vitamin D are solubilized in mixed micelles through the action of bile and pancreatic juices after dissolving in ingested fats and oils. A fraction of the solubilized vitamin D is then absorbed [59]. This study evaluated the intestinal absorption of newly synthesized secosteroids, compounds **3** and **4**, using human intestinal model cells Caco-2 cultured in Transwells. Absorption was defined as cellular uptake and transport from the cell to the basolateral medium, assumed to be the lymph, and compared with vitamin D_3_. Compound **3** showed uptake levels comparable to vitamin D_3_ but had about half the transport efficiency. Conversely, compound **4** displayed higher uptake and equivalent transport compared to vitamin D_3_, indicating its superior or equal intestinal absorption compared to vitamin D_3_. These results can be summarized as compound **4** ≥ vitamin D_3_ ≥ compound **3**, albeit in an intestinal model cell system. The chemical structures of the side chains are different for compound **3**, compound **4**, and vitamin D_3_; the side chains of compound **3** and vitamin D_3_ are the same as those of fucosterol and cholesterol, respectively. However, there is no corresponding sterol for compound **4**. Although we could not find any previous reports on comparative intestinal absorption studies of fucosterol and cholesterol in Caco-2 cells, there was a study of cholesterol and β-sitosterol [60]. According to this study, the uptake and transport by Caco-2 were higher for cholesterol than for sitosterol. The side-chain structures of fucosterol and β-sitosterol are similar, differing only in the presence or absence of a double bond between C24-C24^1^. The absorption behavior of fucosterol was thought to be more similar to that of β-sitosterol than that of cholesterol. The reason why compound **3** was less absorbed than vitamin D_3_ by Caco-2 may be due to the difference in the side-chain structure. In other words, the structure of the side chain seems to be greatly involved in the intestinal absorption of secosteroids.

We previously investigated vitamin D_2_–D_7_ uptake using differentiated Caco-2 cells [49]. Vitamin D_5_ showed lower uptake; however, no significant differences were noted among the other forms of vitamin D. Given that the uptake of compounds **3** and **4** was equal to or higher than that of vitamin D_3_, we expect similar results from comparisons with other forms of vitamin D. However, further studies on transport efficiency are needed.

During incubation, the residual secosteroid in mixed micelles could affect Caco-2 cell absorption. Because other components in the mixed micelles are also absorbed during incubation, secosteroids may aggregate and thus be unabsorbed. If secosteroids do not remain in mixed micelles throughout the incubation, uptake would naturally decrease. Monitoring revealed that the initial concentration of secosteroids in the mixed micelles added to the apical side was 1 μM, implying that more than 30% remained post-incubation. These findings corroborate our earlier studies, indicating sufficient residual secosteroid levels in mixed micelles that did not negatively impact uptake [49,61].

In this study, the potential biological activity of compound **3** as a form of vitamin D was assessed through simulation using PASS online. Vitamin D absorbed in the intestinal tract undergoes metabolic conversion in the liver and kidneys, specifically hydroxylation at the C25/C1 positions, to become activated and functional. Therefore, the structure of compound **3** after such metabolic conversion was subjected to simulation (Figure 5). Its biological activities were then compared to the activated forms of vitamins D_2_–D_7_ (Figure 6).

For basic nutritional functions like bone formation and calcium regulation, compound **3** was estimated to have lower biological activity than vitamins D_2_–D_7_. Although it suggests a relatively reduced nutritional capacity, low activity can sometimes be advantageous. Vitamin D, initially identified as an anti-ricket factor, now has a broader biological activity spectrum. While natural vitamin D poses no issues related to bone and calcium regulation, the activated form could have serious side effects, as discussed later, when administered as a medication. Hence, compound **3** may find utility as a drug with fewer side effects.

The PASS online simulation also estimated high overall activity for all types of vitamin D and compound **3** in treating dermatological conditions (e.g., antipsoriasis, dermatologic). For example, 1,25-di(OH)-vitamin D_3_ has been used therapeutically for psoriasis [62,63], and vitamins D_6_ and D_2_ may treat psoriasis.

Similarly, PASS online predicted high biological activity for vitamin D types and compound **3** in cancer-related applications (e.g., antineoplastic, adenomatous polyposis treatment, apoptosis agonist, antileukemic, and chemopreventive). The relationship between vitamin D_3_ and prostate cancer prevention is well-documented [64,65,66,67,68]. Since some polyps become cancerous, 1,25-di(OH)-vitamins D_5_ and D_4_/D_7_, which have been estimated to have high activity for adenomatous polyposis by the PASS online, have been suggested for colorectal cancer prevention like 1,25-di(OH)-vitamin D_3_ [69,70].

Moreover, previous studies have revealed that 1,25-di(OH)-vitamin D_3_ induced differentiation in leukemia cells [71,72]. 1,25-di(OH)-Vitamins D_4_ and D_7_ reportedly induced differentiation in human HL-60 promyelocytic leukemia cells. At a concentration of 10^−7^ M, 1,25-di(OH)-vitamin D_7_ demonstrated activity approximately two-thirds that of 1,25-di(OH)-vitamin D_3_. 1,25-di(OH)-Vitamin D_4_ showed almost the same activity as 1,25-di(OH)-vitamins D_2_ and D_3_ [73]. Since vitamin D types and compound **3** showed similar antileukemic activities in the PASS online assessment, they may also induce leukemia cell differentiation. Moreover, 1,25-di(OH)-Vitamin D_3_ was previously tested as a potential cancer therapy in several clinical trials [74,75]. Notably, these studies showed side effects like hypercalcemia [75] and hypercalciuria [74].

Furthermore, 1-OH-Vitamin D_5_ has been explored as an alternative 1,25-di(OH)-vitamin D_3_ with fewer side effects. Since vitamin D_5_ had a very low anti-rickets activity in rats, 1-OH-vitamin D_5_ was expected to have a limited ability to absorb calcium in the intestinal tract (thus limiting potential side effects like hypercalcemia). The study found that 1-OH-vitamin D_5_ was as effective as 1,25-di(OH)-vitamin D_3_ at inhibiting human breast cancer cell growth [76]. Additionally, 1-OH-vitamin D_5_ effectively treated chemically induced cancers in mice and rats [77,78,79]. Although less effective than 1,25-di(OH)-vitamin D_3_ [77], 1-OH-vitamin D_5_ features markedly fewer side effects like weight loss and disruptions to calcium levels [80]. Even if vitamins D_5_–D_7_ have low anti-rickets activities in humans, their anti-cancer activities generally carry few side effects. Although compound **3** had the highest Pa value for “chemopreventive”, it may be less capable of basic nutritional functions as well as vitamins D_5_–D_7_ (Table 1). Based on these estimations, compound **3** may also be expected to be a chemotherapeutic agent with fewer side effects.

It is important to highlight that only compound **3** exhibited the output “polarization stimulant” distinct from vitamin D (Table 3). This term signifies a substance that restores the polarized state of cell membranes (negative inside relative to the outside) after depolarization in muscle or nerve fibers. This stimulant likely functions akin to a potassium-ion channel opener. If this holds true, compound **3** might hold therapeutic potential for heart and nerve-related conditions like hypertension, epilepsy, and neuropathic pain, aligning with potassium-ion channel openers’ effects [81,82,83]. Notably, compound **3** demonstrated the highest Pa values for “anti-viral (rhinovirus)” and “anti-fungal” among the compared compounds. Similar to vitamin D’s effectiveness against rhinovirus [84,85,86] and fungal infections such as allergic fungal rhinosinusitis [87] and candidiasis [88], may be mitigated by compound **3**.

Certain diseases (pulmonary tuberculosis, type-1 diabetes, and multiple sclerosis) are linked to sunlight exposure. For instance, vitamin D’s antibacterial mechanism involves inducing the production of the antibacterial peptide cathelicidin, which kills M. tuberculosis [89]. Vitamin D also impacts multiple sclerosis and dementia, such as Alzheimer’s disease [90,91,92,93]. Although PASS online did not generate a Pa value for vitamin D_3_ as a treatment for multiple sclerosis (Table 3), vitamin D_2_ was active in multiple sclerosis treatment and anti-parkinsonian effects. Vitamin D_2_ might be effective for some dementias, as mushroom intake correlates with reduced mild cognitive impairment risk [94]. Notably, the relationship of vitamin D_3_ to antidiabetic (type 1) activity (Table 3) aligns with the sunlight exposure–type 1 diabetes connection [95,96].

These findings are still at the simulation stage. Crucially, the actual metabolic conversion of compounds **3** and **4** to the activated form post-intestinal absorption remains unexplored. Future studies will undoubtedly investigate the metabolic conversion and biological activities of compounds **3** and **4** in cellular and animal experiments. Vitamin D activation is associated with magnesium [97] and iron (central metals in vitamin D hydroxylase) [98]. Deficiencies could hinder vitamin D effects. Intestinal microflora also affect vitamin D activation [99]. Some intervention trials might overlook vitamin D activation issues when observing no effects. Metabolism is a vast research domain in itself.

Although compound **3** was synthesized organically, it could occur naturally. Notably, information about vitamins D_4_–D_7_ in natural products is scarce. Vitamins D_4_ and D_5_ were identified in mushrooms in 2012 [45] and in Arabidopsis mutants in 2018 [100], respectively, and 7-dehydrositosterol, a vitamin D_5_ precursor, was found in red algae [101] and Indian snakewood, which is used medicinally [102,103]. Vitamin D_6_ and D_7_ precursors, 7-dehydrostigmasterol and 7-dehydrocampesterol, respectively, exist in protozoa [104,105]. If UV exposure affects these precursors, vitamins D_6_ and D_7_ could be present. The precursor of compound **3**, 7-dehydrofucosterol, naturally occurs and is found in sponges [106,107,108,109], water molds [110], and protozoa [111]. Exposure to UVB could lead to the production of compound **3**. Future research may uncover 7-dehydrofucosterol’s presence in food and compound **3**’s occurrence in natural products.

## 4. Materials and Methods

### 4.1. Organic Synthesis

#### 4.1.1. Reagents and Conditions for Organic Synthesis

We employed mass spectrometry (MS), nuclear magnetic resonance (NMR), and optical rotation measuring apparatuses to determine the chemical structures of the target compounds and their intermediate products. In positive-ion mode, atmospheric pressure chemical ionization (APCI)-MS data were collected using a Kingdon trap-type mass spectrometer (Orbitrap Veros Pro ETD, Thermo Fisher Scientific, Waltham, MA, USA). Calibration utilized polytyrosine (Pierce^®^ Triple Quadrupole calibration solution, Thermo Fisher Scientific) as an external standard. NMR spectra were acquired with a Bruker BioSpin spectrometer (AV 400, Bruker Corporation, Madison, MA, USA) by dissolving samples in deuterated chloroform (CDCl_3_). Chemical shifts are reported in ppm relative to Me_4_Si (δ 0.00). NMR signal characterizations employed abbreviations: s, singlet; d, doublet; t, triplet; m, multiplet. Optical rotation measurements were performed with a Jasco instrument (P-1020-GT, JASCO Corporation, Tokyo, Japan) using sample solutions in chloroform at room temperature.

UV irradiation for 7-dehydrosterols was accomplished using a Ushio UV lamp (SX-UID 501MAMQQ, Ushio Inc., Tokyo, Japan) in conjunction with a Bunko Keiki spectrometer (UB-100KC, Bunko Keiki Inc., Tokyo, Japan).

For the synthesis reaction product, preparative HPLC was applied to obtain the target compound using the following conditions. HPLC condition 1: Separation was performed on a Waters HPLC system employing a Mightysil RP-18 GP 250-20 column (20 × 250 mm, 5 μm; Kanto Chemical Co., Inc., Tokyo, Japan). An initial isocratic analysis was conducted with ethanenitrile at a flow rate of 1.0 mL/min, followed by a flow rate increase to 5.0 mL/min over 1.0 min starting at 0 min, maintaining this status. Samples were detected at 215 nm. Reagent-grade chemicals and solvents were used.

#### 4.1.2. Organic Synthesis of New Secosteroids

**Figure 7:**
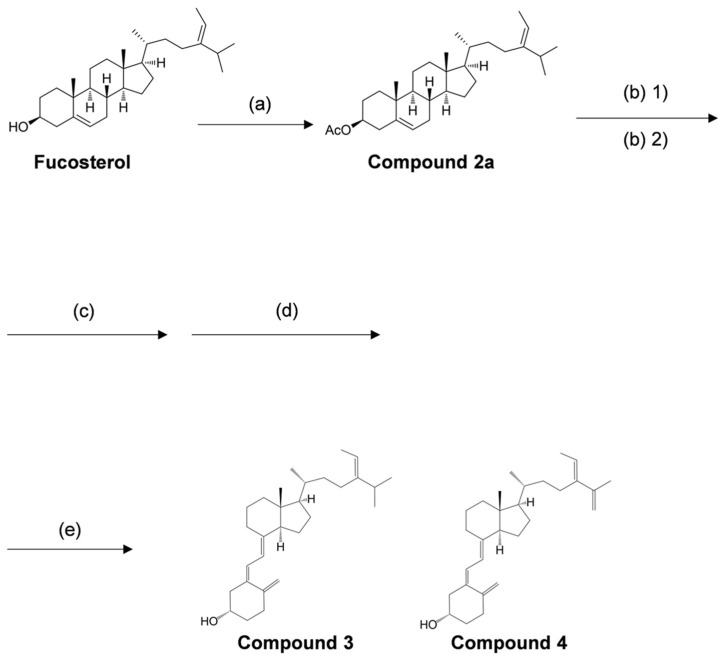
Overview of reagents and conditions employed in various synthesis steps. (**a**) acetic anhydride, pyridine, 45 °C, 3.5 h, 97%. (**b**) (1) N-bromosuccinimide, cyclohexane, 100 °C, 1.5 h, (2) 1.0 M tetra-n-butylammonium fluoride/tetrahydrofuran, room temperature, 12 h, 61%. (**c**) 28% sodium methoxide in methyl alcohol, dichloromethane/methyl alcohol, room temperature, 4 h, 57%. (**d**) 0.1% tert-butyl-4-hydroxyanisole (BHA) in cyclohexane, 280 nm, 9.71 mW/cm², room temperature, 2 h, 11%. (**e**) 0.1% BHA in cyclohexane, 100 °C, 2 h.

The synthetic procedure for the novel secosteroid is outlined in Figure 7. Fucosterol (≥98.0% purity) was obtained from Hairui Chemical Co., Ltd. (Hangzhou, China). Fucosterol (988 mg) was dissolved in 7 mL of pyridine, and 3.0 mL of acetic anhydride was introduced. The mixture was agitated at 45 °C for 3.5 h. Ice was introduced to deactivate the acetic anhydride without elevating the temperature, and the reaction mixture was shaken for 1 h. Following completion, chloroform was added, and 2 N HCl was used to neutralize pyridine in the resulting chloroform layer. Solvent evaporation and recrystallization with ethyl alcohol yielded compound **2a** (1061 mg, 97%). The outcomes were presented sequentially, encompassing optical rotation measurement, NMR measurement, and accurate MS as follows.

[α]^25^_D_ = −45.2 (c = 0.847, chloroform). ^1^H-NMR (400 MHz, CDCl_3_): δ = 0.69 (s, 3H, Me-18), 0.92–1.32 (m, 8H, H-1a, H-9, H-12a, H-14, H-16a, H-17, H-22a, H-23a), 0.978 (d, 3H, J = 6.8 Hz, Me-26 or Me-27), 0.981 (d, 3H, J = 6.8 Hz, Me-26 or Me-27), 0.99 (d, 3H, J = 6.5 Hz, Me-21), 1.02 (s, 3H, Me-19), 1.37–1.64 (m, 8H, H-2a, H-7a, H-11a, H-11b, H-20, H-22b, H-23b, H-25), 1.57 (d, 1H, J = 6.6 Hz, Me-24^2^), 1.82–1.91 (m, 4H, H-1b, H-2b, H-15a, H-16b), 1.94–2.10 (m, 3H, H-7b, H-12b, H-15b), 2.03 (s, 3H, Ac), 2.20 (m, 1H, H-8), 2.32 (m, 2H, H-4a, H-4b), 4.60 (m, 1H, H-3), 5.18 (q, 1H, J = 6.7 Hz, H-24^1^), 5.37 (d, 1H, J = 4.9 Hz, H-6); ^13^C-NMR (100 MHz, CDCl_3_), δ = 11.8 (C-18), 13.2 (C-24^2^), 18.8 (C-21), 19.3 (C-19), 21.0 (C-11), 21.4 (COCH_3_), 22.1 (C-26 or C-27), 22.2 (C-26 or C-27), 24.3 (C-23), 25.7 (C-15), 27.8 (C-2), 28.2 (C-16), 31.9 (C-7 or C-25), 31.9 (C-7 or C-25), 34.8 (C-8), 35.2 (C-22), 36.4 (C-20), 36.6 (C-10), 37.0 (C-1), 38.1 (C-4), 39.7 (C-12), 42.4 (C-13), 50.0 (C-9), 55.8 (C-17), 56.7 (C-14), 74.0 (C-3), 115.6 (C-24^1^), 122.6 (C-6), 139.7 (C-5), 147.0 (C-24), 170.5 (C=O). APCI-Orbitrap-MS: calculated for C_31_ H_51_O_2_^+^ (M + H)^+^: 455.3884, found *m*/*z*: 455.3869.

Compound **2a** (300 mg, 0.66 mmol) was dissolved in 7.5 mL cyclohexane at 65 °C. N-Bromosuccinimide (NBS) (176 mg, 0.989 mmol) was added, and the reaction mixture was stirred under reflux conditions (100 °C) for 1.5 h. The reaction temperature was lowered to room temperature, and 15 mL of water was added. The mixture was stirred at room temperature for an additional 1 h. The reactants were extracted with normal hexane and washed with water, and the solvent was removed under vacuum. The resulting dry product was dissolved in 1.5 mL of 1 M tetrabutylammonium fluoride tetrahydrofuran (Bu_4_NF/THF) solution and stirred at room temperature for 12 h. The reaction mixture was extracted with normal hexane, and the extract was washed with water and concentrated in vacuo. The product was subjected to silica column chromatography and eluted with acetic ether/normal hexane (1:30, *v*/*v*), yielding a fraction containing the compound with a UV absorption region displaying a 5,7-diene structure (184 mg).

The total amount of the obtained compound was dissolved in 2 mL of dichloromethane and 5 mL of methyl alcohol. To this mixture, 28% sodium methylate (NaOMe) methyl alcohol solution was added until the pH reached 12. The mixture was stirred at room temperature for 4 h. The reaction solvent was concentrated, and the product was subjected to silica column chromatography, eluting with acetic ether/normal hexane (3:7, *v*/*v*), yielding a fraction containing the deacetylated compound (104.31 mg).

The obtained compound (95.31 mg) was dissolved in 25 mL of cyclohexane containing 0.1% 3-tert-butyl-4-hydroxyanisole (BHA). The entire volume was transferred to a Petri dish covered with polyvinylidene chloride food wrap. The reaction was irradiated with 280 nm UV light (9.71 mW/cm²) for 2 h at room temperature while stirring with a magnetic stirrer. After irradiation, the reaction solvent was concentrated, and the product was subjected to silica column chromatography and eluted with acetic ether/normal hexane (2:8, *v*/*v*), forming the irradiated compound (10.71 mg). This compound was dissolved in 10 mL of cyclohexane containing 0.1% BHA and stirred under reflux conditions (100 °C) for 2 h. The reaction solvent was concentrated and subjected to silica column chromatography, eluting with acetic ether/normal hexane (2:8, *v*/*v*), to yield a mixture of compounds **3** and **4** (single spot on TLC). The obtained mixture was further subjected to HPLC condition 1 as previously described. The two pure compounds, **3** (4.10 mg, retention time at 75.4 min) and **4** (1.13 mg, retention time at 60.1 min), were isolated. The outcomes were presented sequentially, encompassing optical rotation measurement, NMR measurement, and accurate MS as follows.

(5*Z*,7*E*,24*E*)-(3S)-9,10-seco-5,7,10(19),24(24^1^)-stigmastatetraen-3-ol (Compound **3**); [α]^25^_D_ = +25.6 (c = 0.098, chloroform); ^1^H-NMR (400 MHz, CDCl_3_), δ = 0.55 (s, 3H, Me-18), 0.979 (d, 3H, J = 6.7 Hz, Me-26 or Me-27), 0.983 (d, 3H, J = 6.8 Hz, Me-26 or Me-27), 0.99 (d, 3H, J = 5.2 Hz, Me-21), 1.06–1.17 (m, 1H, H-22a), 1.26–1.42 (m, 5H, H-12a, H-16a, H-17, H-20, H-22b), 1.46–1.72 (m, 6H, H-2a, H-9a, H-11a, H-11b, H-15a, H-15b), 1.57 (d, 3H, J = 6.9 Hz, Me-24^2^), 1.83–2.11 (m, 6H, H-2b, H-12b, H-14, H-16b, H-23a, H-23b), 2.14–2.23 (m, 2H, H-1a, H-25), 2.29 (dd, 1H, J = 13.3, 7.6 Hz, H-4a), 2.40 (m, 1H, H-1b), 2.57 (dd, 1H, J = 13.0, 3.8 Hz, H-4b), 2.82 (m, 1H, H-9b), 3.95 (m, 1H, H-3), 4.82 (d, 1H, J = 2.4 Hz, H-19a), 5.05 (broad s, 1H, H-19b), 5.19 (q, 1H, J = 6.7 Hz, H-24^1^), 6.03 (d, 1H, J = 11.3 Hz, H-7), 6.24 (d, 1H, J = 11.2 Hz, H-6); ^13^C-NMR (100 MHz, CDCl_3_), δ = 12.0 (C-18), 13.2 (C-24^2^), 18.9 (C-21), 22.1 (C-15 or C-26 or C-27), 22.2 (C-15 or C-26 or C-27), 22.3 (C-15 or C-26 or C-27), 23.6 (C-11), 25.7 (C-23), 27.7 (C-16), 29.0 (C-9), 31.9 (C-1), 34.8 (C-25), 35.2 (C-2 and C-22), 36.7 (C-20), 40.5 (C-12), 45.87 (C-4 or C-13), 45.92 (C-4 or C-13), 56.25 (C-14 or C-17), 56.33 (C-14 or C-17), 69.2 (C-3), 112.4 (C-19), 115.6 (C-24^1^), 117.5 (C-7), 122.5 (C-6), 135.1 (C-5), 142.3 (C-8), 145.1 (C-10), 147.0 (C-24); NOESY-NMR (400 MHz, CDCl_3_), δ = 5.19 (H-24^1^) → 0.979 or 0.983 (Me-26), 5.19 (H-24^1^) → 1.57 (Me-24^2^), 5.19 (H-24^1^) → 2.14–2.23 (H-25); APCI-Orbitrap-MS, calculated for C_29_H_47_O^+^ (M + H)^+^: 411.3621, found *m*/*z*: 411.3620.

(5*Z*,7*E*,24*E*)-(3*S*)-9,10-seco-5,7,10(19),24(24^1^),25(27)-stigmastapentaen-3-ol (Compound **4**); [α]^25^_D_ = +8.9 (c = 0.023, chloroform); ^1^H-NMR (400 MHz, CDCl_3_), δ = 0.55 (s, 3H, Me-18), 1.02 (d, 3H, J = 6.4 Hz, Me-21), 1.13 (m, 1H, H-22a), 1.23–1.35 (m, 3H, H-12a, H-16a, H-17), 1.38–1.59 (m, 5H, H-11a, H-15a, H-15b, H-20, H-22b), 1.64–1.72 (m, 3H, H-2a, H-9a, H-11b), 1.71 (d, 3H, J = 6.9 Hz, Me-24^2^), 1.85–2.02 (m, 4H, H-2b, H-12b, H-14, H-16b), 1.87 (d, 1H, J = 0.8 Hz, Me-26), 2.08–2.21 (m, 2H, H-1a, H-23a), 2.26–2.43 (m, 3H, H-1b, H-4a, H-23b), 2.57 (dd, 1H, J = 13.0, 3.7 Hz, H-4b), 2.82 (m, 1H, H-9b), 3.95 (m, 1H, H-3), 4.82 (d, 1H, J = 2.5 Hz, H-19a), 4.86 (s, 1H, H-27a), 4.96 (s, 1H, H-27b), 5.05 (broad s, 1H, H-19b), 5.63 (q, 1H, J = 6.9 Hz, H-24^1^), 6.03 (d, 1H, J = 11.3 Hz, H-7), 6.24 (d, 1H, J = 11.3 Hz, H-6); ^13^C-NMR (100 MHz, CDCl_3_), δ = 12.0 (C-18), 13.9 (C-24^2^), 18.9 (C-21), 21.3 (C-26), 22.3 (C-15), 23.6 (C-11), 24.2 (C-23), 27.7 (C-16), 29.0 (C-9), 31.9 (C-1), 35.0 (C-2 or C-22), 35.2 (C-2 or C-22), 36.7 (C-20), 40.5 (C-12), 45.87 (C-4 or C-13), 45.92 (C-4 or C-13), 56.2 (C-14 or C-17), 56.3 (C-14 or C-17), 69.2 (C-3), 110.3 (C-27), 112.4 (C-19), 117.5 (C-7), 121.6 (C-24^1^), 122.5 (C-6), 135.1 (C-5), 141.3 (C-24), 142.2 (C-8), 143.6 (C-25), 145.1 (C-10); NOESY-NMR (400 MHz, CDCl_3_), δ = 5.63 (H-24^1^) → 1.71 (Me-24^2^), 5.63 (H-24^1^) → 1.87 (Me-26), 4.86 (H-27a) → 1.87 (Me-26), 4.96 (H-27b) → 2.08–2.21 (H-23a), 4.96 (H-27b) → 2.26–2.43 (H-23b); APCI-Orbitrap-MS, calculated for C_29_H_45_O^+^ (M + H)^+^: 409.3465, found *m*/*z*: 409.3465.

### 4.2. Cell Biochemistry

#### 4.2.1. Materials

Vitamin D_3_ (also known as cholecalciferol, >98.0% purity) was purchased from Tokyo Chemical Industry Co., Ltd. (Tokyo, Japan). Trolox, vitamin K_1_ (phylloquinone), sodium taurocholate, oleic acid, 1-oleoyl-rac-glycerol (oleoylglycerol), 1-16:0-2-OH-sn-glycerol-3-phosphocholine (lysoPC), and fetal bovine serum (FBS) were purchased from Sigma-Aldrich (St. Louis, MO, USA). Three types of Dulbecco’s Modified Eagle’s Medium (DMEM, low-glucose type) were purchased from Nissui Pharmaceutical (with phenol red and without L-glutamine, Tokyo, Japan), Sigma-Aldrich (without phenol red and with L-glutamine), and Sigma-Aldrich (without both phenol red and L-glutamine). Nonessential amino acids (NEAA), penicillin, streptomycin, and amphotericin B were purchased from Thermo Fisher Scientific. The other chemicals and solvents were of reagent grade.

#### 4.2.2. Cells and Culture

Caco-2 human colorectal adenocarcinoma cell lines were obtained from the ATCC (HTB-37, Rockville, MD, USA). FBS was heat-inactivated at 56 °C for 30 min. Subculture medium containing DMEM with 4 mM L-glutamine, 40 units/mL of penicillin, 40 µg/mL of streptomycin, 0.1 mM NEAA, and 10% FBS was used for cell growth. The cells were cultured at 37 °C in a humidified atmosphere with 5% CO_2_. The passage of cells was performed twice weekly.

#### 4.2.3. Human Intestinal Cell Model

Caco-2 cells were differentiated through continuous medium exchange over approximately 3 weeks to establish a human intestinal model. The differentiation process followed a previous method [61]. In brief, subcultured cells were seeded into the polycarbonate insert membranes of Transwell^®^ 6-well plates (Corning Inc., Kennebunk, ME, USA) at a density of 3 × 10^5^ cells/well. The medium was changed twice a week for 28 days at 37 °C in a humidified atmosphere with 5% CO_2_. The differentiation medium used for medium exchange consisted of DMEM (Nissui) with 4 mM L-glutamine, 40 units/mL of penicillin, 40 µg/mL of streptomycin, 0.1 µg/mL of amphotericin B, 0.1 mM NEAA, and 10% FBS. Transepithelial electrical resistance (TEER) was measured using a voltohmmeter equipped with a chopstick-type electrode (Millicell ERS-2 and MERS STX01; Merck, Burlington, MA, USA) before the absorption experiment. TEER values were approximately 300 Ω, indicating the formation of tight monolayers, consistent with a prior report [112].

#### 4.2.4. Mixed Micelles Preparation

Mixed micelles were prepared following a previous report [49]. Each component of the mixed micelles (lysoPC, oleic acid, oleoylglycerol, secosteroid, and taurocholate) was dissolved in an appropriate solvent. A portion of each solution was placed in glass test tubes. The solvent was removed under a stream of inert argon gas, and the solution was completely dried using a centrifugal evaporator. The resulting material was dispersed in DMEM (Nissui) with 4 mM L-glutamine, 40 units/mL of penicillin, 40 µg/mL of streptomycin, 0.1 µg/mL of amphotericin B, and 0.1 mM NEAA. This medium was equivalent to the differentiation medium without FBS. The dispersed solution was passed through a 0.2 µm filter to eliminate any unsolubilized secosteroid. The filtrate, contained lysoPC (50 µM), oleic acid (33 µM), oleoylglycerol (100 µM), secosteroid (1 µM), and taurocholate (2 mM), were used as the mixed micelles (apical-side medium). These component concentrations matched those in a previous study [49]. In addition, the past literature was also referred to regarding the set concentration of secosteroids [113].

The initial secosteroid concentration was determined before introducing mixed micelles to Caco-2 cells. A portion of the mixed micelles was diluted fourfold with dichloromethane/methyl alcohol (1:4, *v*/*v*), and then a portion (30 µL) underwent HPLC condition 2 as described below. The initial concentration for all experiments was determined to be 1.01 ± 0.02 µM.

#### 4.2.5. Evaluation of Secosteroid Intestinal Absorption (Uptake and Transport)

Both apical and basolateral sides of the tight monolayers in Transwell plates were washed twice with a washing medium comprising DMEM (Sigma-Aldrich, without phenol red and L-glutamine) with 40 units/mL of penicillin, 40 µg/mL of streptomycin, and 0.1 mM NEAA. Subsequently, 1.5 mL of the mixed micelles prepared earlier (apical-side medium) and 2.5 mL of basal-side medium consisting of DMEM (Sigma-Aldrich, without phenol red and with L-glutamine) with 40 units/mL of penicillin, 40 µg/mL of streptomycin, 0.1 µg/mL of amphotericin B, 0.1 mM NEAA, and 10% FBS were added to their respective sides. The plates were then incubated at 37 °C with 5% CO_2_ for 24 h. Following incubation, the next steps were to assess (i) transported secosteroid quantity through the monolayers to the basolateral side, (ii) secosteroid uptake by cells, and (iii) residual secosteroid levels in the mixed-micellar apical-side medium. For the extraction of secosteroid, 1 µM phylloquinone/ethyl alcohol with 2 µM trolox as an antioxidant served as the internal standard, following the same procedure as for (i)–(iii).

(i): Initially, 20 µL of 0.2 mM trolox/ethyl alcohol was added to the collected basal-side medium (approximately 2.5 mL). Then, the obtained mixture (2.2 mL) was combined with the internal standard (0.2 mL). Subsequently, ethyl alcohol (2.0 mL), acetic ether (2.2 mL), and normal hexane (2.2 mL) were added, with the solution shaken using a Vortex mixer after each addition. The upper phase of the two-layered solution was collected, and the bottom phase underwent the same procedure with the addition of acetic ether and normal hexane, followed by shaking. The upper phases were combined, and the phase was dried using a centrifugal evaporator. The resulting extract was dissolved in 200 µL of dichloromethane/methyl alcohol/water (38:152:10, *v*/*v*/*v*), with an aliquot (60 µL) submitted to HPLC condition 2, described below.
  In the event of any disruption to the tight monolayer due to an experimental mishap, the target component (in this case, secosteroid) could leak from the apical to the basolateral side. Phenol red in the apical-side medium was used to indicate such leakage. After incubation, phenol red concentration in the collected basolateral medium (not present initially) was measured at 560 nm under alkaline conditions. The Tecan Infinite F50R microplate reader (Tecan Group Ltd., Männedorf, Switzerland) was used for this measurement.
(ii): The differentiated Caco-2 cells, forming the tight monolayers, were rinsed twice with Hank’s balanced salt solution. These cells were gathered in glass tubes by gently detaching them from the polycarbonate membrane of the insert wells using the back of a spatula. The purpose was to measure the cellular uptake of secosteroids. The cells were homogenized in 2.0 mL of PBS containing 20 µL of 0.2 mM trolox/ethyl alcohol using a probe-type sonicator (Ultra S, VP-5S; Taitec, Saitama, Japan). A portion (1.8 mL) of the cell homogenate suspension was mixed with 0.2 mL of the internal standard. Following this, 1.6 mL of ethyl alcohol, 1.8 mL of acetic ether, and 1.8 mL of normal hexane were introduced. Subsequent extraction and drying steps were performed as described in i). The resulting extract was dissolved in 200 µL of dichloromethane/methyl alcohol/water (38:152:10, *v*/*v*/*v*). This solution’s aliquot (20 µL) was submitted for HPLC under condition 2, described below.
  The protein content of the cells was assessed using the DC protein assay kit (Bio-Rad Laboratories, Hercules, CA, USA). An aliquot (50 µL) of the cell homogenate suspension was diluted fourfold in PBS and applied to the kit. The final reaction solution’s absorbance was measured at 750 nm using the same microplate reader mentioned earlier. Cellular uptake of secosteroid was normalized based on this protein level.
(iii): The reduction in micellar secosteroid due to aggregation might have influenced secosteroid uptake from mixed micelles. This is because only micellar secosteroids could be assimilated by Caco-2 cells, as explained in our prior report [49].

Around 1.5 mL of the apical-side medium was collected and augmented with 20 µL of 0.2 mM trolox/ethyl alcohol. Approximately 1.0 mL of the collected medium underwent filtration as previously described. A portion (0.8 mL) was mixed with 0.2 mL of the internal standard. Subsequently, 0.6 mL of ethyl alcohol, 0.8 mL of acetic ether, and 0.8 mL of normal hexane were added, and the subsequent extraction and drying processes followed the same steps as outlined in i). The resulting extract was dissolved in 400 µL of dichloromethane/methyl alcohol/water (38:152:10, *v*/*v*/*v*), with an aliquot (20 µL) submitted for HPLC under condition 2.

Recoveries (%, *n* = 4) of the secosteroid spiked into the apical-side medium, cellular suspension of differentiated Caco-2, and basal-side medium were 95.9 ± 0.9, 94.9 ± 1.3, and 97.0 ± 1.5, respectively.

#### 4.2.6. HPLC Analysis (HPLC Condition 2)

Secosteroid analysis was conducted using a semi-micro HPLC system (Shimadzu, Kyoto, Japan) equipped with a pump (LC-20AT), a photodiode array detector (SPD-M10A), and a column oven (CTO-10AS) set at 25 °C on an ODS-80Ts column (2.0 × 150 mm; Tosoh, Tokyo, Japan) with an ODS-S1 precolumn (2.0 × 10 mm; Tosoh). An isocratic analysis was performed at a flow rate of 0.2 mL/min using an ethanenitrile/methyl alcohol/acetic ether/water (45:107:42:6, *v*/*v*/*v*/*v*) mixture containing 0.1% acetic acid ammonium. The quantification of secosteroids relied on the peak area at 265 nm, utilizing calibration curves of the standard.

All experiments were conducted under subdued yellow light to mitigate the isomerization and degradation of secosteroids induced by light exposure.

#### 4.2.7. Statistical Analysis

We analyzed the data nonparametrically by using the Kruskal–Wallis test, and the significant differences of means were evaluated by the Mann–Whitney U-test. *p*-Values less than 0.05 were considered statistically significant. The statistical analyses were performed using StatView for Windows version 5.0 (SAS Institute Inc., Cary, NC, USA).

### 4.3. Online Simulation for Estimating Biological Activities of Secosteroids

The biological activity of the organically synthesized secosteroid, resembling vitamin D, was projected using PASS online simulation (http://www.pharmaexpert.ru/passonline/, accessed on 12 May 2023). While vitamin D is inactive, its activated form is generated by introducing OH groups at positions C25 and C1 in the liver and kidneys, respectively. This activated form is responsible for its function. Hence, the biological activity of the activated form was estimated, akin to our prior investigations with vitamins D_2_–D_7_ [49].

Results were provided in Pa value (higher values imply greater activity) and Pi value (higher values indicate greater inactivity). A Pa value exceeding 0.7 indicates a high likelihood of experimental biological activity, while Pa values ranging from 0.5 to 0.7 suggest a low likelihood of experimental biological activity, though the compound might differ from known drugs [114]. Organically synthesized secosteroid biological activities were juxtaposed with those of vitamins D_2_–D_7_.

## 5. Conclusions

We synthesized novel secosteroids—compounds **3** and **4**—using fucosterol as the starting material through organic synthesis. To evaluate their potential for intestinal absorption, we conducted tests using an intestinal Caco-2 cell model system and compared their absorption levels with those of vitamin D_3_. The assessment of intestinal absorption encompassed both cellular uptake and cell-to-basolateral transport measurements. Notably, the absorption amount of compound **4** was similar to vitamin D_3_. In the case of compound **3**, its uptake amount mirrored that of vitamin D_3_. Although the transport amount was approximately half of that observed for vitamin D_3_, it was still evident that absorption occurred within the cell model. After intestinal absorption, vitamin D exerts its biological activity through metabolic conversion to an activated form. With this in mind, we subjected the activated forms of the compounds to PASS online simulation to predict their potential biological activity. A Pa value closer to 1.0 indicates higher biological activity, with a Pa value of 0.7 or more suggesting significant activity. While the trophic biological activity of compound **3** in regulating bone and calcium control was found to be comparatively lower than vitamins D_2_–D_7_, its Pa value remained generally high. Similarly, regarding biological activity relating to skin disease therapeutic effects and anti-cancer actions, compound **3** exhibited Pa values on par with vitamins D_2_–D_7_. Interestingly, there were certain items in which compound **3** displayed the highest Pa value among the group. It is worth noting that an attribute unique to compound **3** emerged, suggesting potential biological activity not present in vitamins D_2_–D_7_. We also delved into the prospect of compound **3** occurring naturally. The 7-dehydro form, a precursor to compound **3**, exists in natural products, and exposure to UVB radiation may lead to the natural generation of compound **3**. Comprehensive investigations into the metabolic conversion of compounds **3** and **4** to their activated forms and pragmatic functional studies using cellular and animal models constitute promising avenues for future research. If compound **3** does indeed have nutritional functions related to bone health and calcium regulation, like vitamins D_2_–D_7_, it could potentially be called “fucocalciferol”.

## Figures and Tables

**Table 1:** Predicted biological activities for 1, 25-di(OH) forms of vitamin D and compound **3**, part 1.

	Vitamin D_2_		Vitamin D_3_		Vitamin D_4_/D_7_ *		Vitamin D_5_		Vitamin D_6_		Compound 3 **	
Activity	Pa ^1^	Pi ^2^	Pa	Pi	Pa	Pi	Pa	Pi	Pa	Pi	Pa	Pi
Anti-osteoporotic	0.982 ^a^	0.003	0.973 ^b^	0.003	0.969	0.003	0.965	0.003	0.970	0.003	0.940	0.003
Bone diseases treatment	0.981 ^a^	0.003	0.976 ^b^	0.003	0.968	0.003	0.966	0.003	0.966	0.003	0.938	0.003
Vitamin	0.977 ^b^	0.000	0.975	0.000	0.936	0.000	0.978 ^a^	0.000	0.976	0.000	0.907	0.000
Hyperparathyroidism treatment	0.946 ^a^	0.000	0.933 ^b^	0.000	0.883	0.000	0.882	0.000	0.901	0.000	0.846	0.000
Calcium regulator	0.902 ^a^	0.001	0.876	0.002	0.869	0.002	0.870	0.002	0.880 ^b^	0.001	0.821	0.002
Vitamin D-like	0.869 ^a^	0.000	0.757	0.000	0.578	0.000	0.791	0.000	0.844 ^b^	0.000	0.570	0.000
Vitamin D receptor agonist	0.816 ^a^	0.000	0.693	0.000	0.680	0.000	0.686	0.000	0.738 ^b^	0.000	0.542	0.000

^1, 2^ The values of Pa (probability to be active) and Pi (probability to be inactive) were output by Prediction of Activity Spectra for Substances (PASS) online. Pa values closer to 1 are more active, while Pi values represent inactivity. * These isomers are distinguished by PASS. ** Compound **3** may be applicable. Compound **4** remains uncertain. ^a^ Highest activity among those tested. ^b^ Second-highest activity among those tested. a and b were determined from both Pa and Pi values.

**Table 2:** Predicted biological activities for 1,25-di(OH) forms of vitamin D and compound **3**, part 2.

	Vitamin D_2_		Vitamin D_3_		Vitamin D_4_/D_7_		Vitamin D_5_		Vitamin D_6_		Compound 3	
Activity	Pa	Pi	Pa	Pi	Pa	Pi	Pa	Pi	Pa	Pi	Pa	Pi
Antipsoriatic	0.987 ^b^	0.002	0.976	0.002	0.959	0.002	0.962	0.002	0.992 ^a^	0.001	0.938	0.002
Dermatologic	0.985 ^b^	0.002	0.975	0.003	0.963	0.003	0.966	0.003	0.988 ^a^	0.002	0.950	0.003
Anti-eczematic	0.948	0.003	0.953 ^a^	0.002	0.947	0.003	0.948	0.003	0.946	0.003	0.949 ^b^	0.003
Antipruritic	0.800	0.004	0.834 ^a^	0.002	0.824	0.003	0.825 ^b^	0.003	0.791	0.004	0.821	0.003
Antineoplastic	0.925 ^b^	0.005	0.886	0.005	0.861	0.006	0.873	0.005	0.936 ^a^	0.004	0.909	0.005
Adenomatous polyposis treatment	0.840	0.002	0.894	0.001	0.905 ^b^	0.001	0.916 ^a^	0.001	0.852	0.002	0.877	0.001
Apoptosis agonist	0.782 ^a^	0.009	0.742	0.011	0.642	0.021	0.665	0.019	0.765 ^b^	0.010	0.718	0.013
Antileukemic	0.609 ^a^	0.008	0.599	0.009	0.591	0.009	0.601 ^b^	0.009	0.558	0.010	0.597	0.009
Chemopreventive	0.861	0.003	0.873	0.003	0.844	0.003	0.874 ^b^	0.003	0.868	0.003	0.917 ^a^	0.002

^a^ Highest activity among those tested. ^b^ Second-highest activity among those tested.

**Table 3:** Predicted biological activities for 1,25-di(OH) forms of vitamin D and compound **3**, part 3.

	Vitamin D_2_		Vitamin D_3_		Vitamin D_4_/D_7_		Vitamin D_5_		Vitamin D_6_		Compound 3	
Activity	Pa	Pi	Pa	Pi	Pa	Pi	Pa	Pi	Pa	Pi	Pa	Pi
Respiratory analeptic	0.828	0.007	0.962 ^a^	0.003	0.931	0.004	0.961 ^b^	0.003	0.843	0.006	0.940	0.004
Analeptic	0.714	0.009	0.924 ^a^	0.003	0.840	0.004	0.877 ^b^	0.004	0.738	0.008	0.853	0.004
Anti-inflammatory	0.700	0.016	0.747 ^a^	0.010	0.660	0.021	0.699	0.016	0.688	0.017	0.736 ^b^	0.012
Antidiabetic (type 1)	0.598	0.002	0.824 ^a^	0.001	0.630	0.002	0.635 ^b^	0.002	0.566	0.002	0.592	0.002
Polarization stimulant	- *	-	-	-	-	-	-	-	-	-	0.870 ^a^	0.001
Multiple sclerosis treatment	0.631 ^a^	0.005	-	-	0.296	0.059	0.312	0.050	0.380 ^b^	0.029	-	-
Anti-parkinsonian, rigidity relieving	0.626 ^a^	0.004	0.445	0.013	0.439	0.013	0.439	0.013	0.595 ^b^	0.004	0.379	0.026
Anti-viral (Rhinovirus)	0.480	0.032	0.549 ^b^	0.012	0.538	0.014	0.538	0.014	0.477	0.034	0.591 ^a^	0.007
Anti-fungal	0.445	0.040	0.521	0.027	0.549	0.024	0.606 ^b^	0.018	0.474	0.035	0.628 ^a^	0.016

^a^ Highest activity among those tested. ^b^ Second-highest activity among those tested. * No output; not applicable.

## Data Availability

The data presented in this study are available on request to the corresponding author by contacting the National Agricultural Research Organization website (www.naro.go.jp/english/inquiry/index.html).
